# A multicenter study of severity and prognosis of symptomatic COVID‐19 in end‐stage renal disease and non‐dialysis patients in East of Iran

**DOI:** 10.1002/iid3.1188

**Published:** 2024-03-08

**Authors:** Tara Mohsenzadeh, Masood Ziaee, Hamid Salehiniya, Hossein Mohsenzadeh, Amin Mirsani, Vajehallah Raeesi

**Affiliations:** ^1^ Department of Medicine Birjand University of Medical Sciences Birjand Iran; ^2^ Department of Infectious Diseases, School of Medicine, Infectious Diseases Research Center Birjand University of Medical Sciences Birjand Iran; ^3^ Department of Epidemiology and Biostatistics, School of Health, Social Determinants of Health Research Center Birjand University of Medical Sciences Birjand Iran; ^4^ Department of Pediatrics Mashhad University of Medical Sciences Mashhad Iran; ^5^ Department of Medicine Gonabad University of Medical Sciences Gonabad Iran; ^6^ Department of Internal Medicine, School of Medicine Birjand University of Medical Sciences Birjand Iran

**Keywords:** COVID‐19, dialysis, end‐stage renal disease, prognosis

## Abstract

**Objectives:**

This study aimed to assess the severity and related factors of symptomatic COVID‐19 in end‐stage renal disease (ESRD) patients from several centers in Eastern Iran.

**Methods:**

In this retrospective cohort study, after obtaining ethical approval, 410 patients diagnosed with COVID‐19 were included for analysis. Patients were categorized into two groups based on their dialysis status: the dialysis group (ESRD patients undergoing hemodialysis) and the non‐dialysis group (those without chronic dialysis). Demographic information, clinical symptoms, laboratory tests at admission, length of hospitalization, ICU admission, need for mechanical ventilation, and mortality data were extracted from their medical records and entered into researcher‐developed checklists.

**Results:**

In this multicenter study, 104 dialysis patients with a mean age of 64.81 ± 16.04 were compared to 316 non‐dialysis patients with a mean age of 60.92 ± 17.89. Patients were similar in terms of age and gender, but a higher percentage of the dialysis group was aged over 65 years (*p* = .008). Altered consciousness, dyspnea, headache, myalgia, anorexia, and cough were statistically significantly more common in the dialysis group when evaluating clinical symptoms (*p* < .05). The dialysis group had significantly higher levels of white blood cell (WBC), potassium, calcium, urea, creatinine, blood pH, INR, ALT, ESR, and CRP, and lower levels of red blood cell, Hb, platelets, sodium, and LDH compared to the non‐dialysis group. Profoundly altered consciousness was more common among deceased patients (*p* < .001), and this group had higher WBC counts, urea levels, AST, ALT (*p* < .05), and lower blood pH (*p* = .001).

**Conclusion:**

Based on the results of this study, it is plausible to suggest a hypothesis of greater severity and worse prognosis of COVID‐19 in ESRD patients. Underlying comorbidities, such as liver disorders or more severe clinical symptoms like altered consciousness, may also be indicative of a worse prognosis in dialysis patients with COVID‐19.

## Graphical abstract

Based on the results of this study, it is plausible to suggest a hypothesis of greater severity and worse prognosis of COVID‐19 in end‐stage renal disease patients. Underlying comorbidities, such as liver disorders or more severe clinical symptoms like altered consciousness, may also be indicative of a worse prognosis in dialysis patients with COVID‐19.

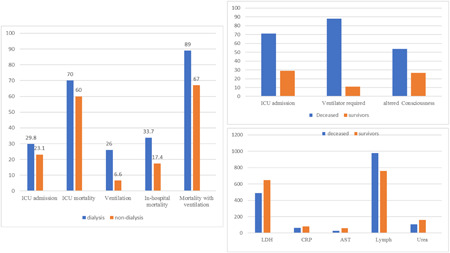

## 1 INTRODUCTION

Severe acute respiratory syndrome coronavirus 2 (SARS‐CoV‐2) is one of the new members of the Beta‐coronavirus RNA family and is the causative agent of severe pneumonia.^1^ The hallmark of SARS‐CoV‐2 pathogenesis is the release syndrome, characterized by an uncontrolled and lethal systemic inflammatory response resulting from the excessive secretion of proinflammatory cytokines, including granulocyte colony‐stimulating factor (G‐CSF), interferon gamma‐inducible protein 10 (CXCL10 or IP10), monocyte chemoattractant protein‐1 (MCP1), macrophage inflammatory protein‐1a (MIP1A), IL‐2, IL‐6, IL‐7, and TNF.^2^, ^3^ Cytokines released in COVID‐19 have been associated with lung edema, impaired gas exchange, acute cardiac injury, acute respiratory distress syndrome, and mortality. Biochemical tests have also identified diagnostic markers such as elevated LDH, liver enzymes AST and ALT, and decreased albumin. Total bilirubin and creatinine levels have also been recognized as predictive factors in COVID‐19 patients.^4^

The kidneys may be secondary targets of SARS‐CoV‐2 after the respiratory system. Viral RNA loads have been detected in kidney tissues postmortem following COVID‐19‐related deaths.^5^ The exact mechanisms underlying kidney involvement in COVID‐19 are still not fully understood, but kidneys may be more sensitive to inflammation or immune system activation. Furthermore, the coagulation abnormalities often seen in COVID‐19 patients could potentially disrupt kidney function.^6^ The significantly high viral load in severe COVID‐19 could be due to the rich expression of genes encoding proteins such as angiotensin‐converting enzyme 2 (ACE2), membrane serine protease 2 (TMPRSS2), and cathepsin L (CTSL) in kidney glomeruli.

Moreover, chronic kidney disease (CKD) has been associated with the severity and mortality of COVID‐19. Kidney problems can persist for months after recovery from the initial coronavirus infection, leading to a decline in kidney function in some patients.^7^ CKD is characterized by irreversible kidney function impairment and may progress to end‐stage renal disease (ESRD), requiring alternative treatments such as dialysis or kidney transplantation. ESRD accounts for nearly 70% of all cases of CKD.^8^ Statistics indicate that ESRD patients have a threefold increased risk of severe COVID‐19 and about one‐third of those hospitalized with COVID‐19 die.^9^ Hospitalized ESRD patients require more ventilator support, and exhibit higher levels of blood urea, and elevated ferritin. Commonly reported symptoms in these patients include lethargy, anorexia, which is often associated with uremic syndrome, dry cough, fever, dyspnea, higher lymphocyte counts, and increased procalcitonin levels.^10^ Among hemodialysis patients, fever and cough are less common. Therefore, the absence of fever may pose challenges in early diagnosis in some hemodialysis patients. Overall, hemodialysis patients exhibit more prominent laboratory abnormalities, chest scan findings, and weaker clinical outcomes.^10^

Independent risk factors for in‐hospital mortality in patients with ESRD included older age, the use of mechanical ventilation, lymphopenia, elevated blood urea nitrogen (BUN), serum ferritin, lymphocyte count, and cytokine storm.^11^, ^12^

According to studies, chronic hemodialysis patients chronically have relatively higher levels of inflammatory markers^13^ and exhibit lower levels of these markers during the cytokine storm response to COVID‐19.^14^ Additionally, due to the dysfunction of the renin‐angiotensin system in dialysis patients, they have less lung involvement.^15^ Furthermore, these individuals have fewer T cells, B cells, and natural killer cells due to immune system deficiencies.^14^

Kazmi et al. found that hypoalbuminemia, leukocytosis, lymphopenia, and elevated LDH were associated with mortality in ESRD patients with COVID‐19, with percentages of 81.8%, 72.7%, 100%, and 100%, respectively.^16^ Rastad et al. showed that COVID‐19 patients with ESRD had an in‐hospital mortality rate of 37.8% compared to 11.9% for those without ESRD. In the ESRD subgroup, individuals who died were older and likely presented with altered mental status or oxygen saturation <93%. They also had lower lymphocyte counts but higher neutrophil counts and AST levels.^17^

Naaraayan et al. indicated that COVID‐19 patients with ESRD had a milder course of the disease, a lower likelihood of severe pneumonia, and a reduced need for invasive oxygen.^18^ Turgutalp et al. demonstrated that elevated AST levels, low platelet counts, high LDH levels, anemia, leukopenia, and thrombocytopenia were associated with morbidity and mortality in these patients.^19^

Considering the contradictions regarding the severity and influencing factors of COVID‐19 in ESRD patients, further studies in this area are needed. On the other hand, the unique pathophysiology of hemodialysis patients highlights the need for judicious monitoring in these subgroups of patients based on treatment protocols in each country. Since there have been limited studies investigating the severity of COVID‐19 in ESRD patients in our country, this current study aimed to determine the severity and prognosis of symptomatic COVID‐19 in ESRD (dialysis) patients compared to non‐dialysis patients.

## 2 METHOD

### 2.1 Study design and setting

The research employed a retrospective cohort study design and focused on COVID‐19 patients receiving medical care across multiple state hospitals in two distinct provinces in Iran: South Khorasan (cities: Birjand, Ferdows, and Gaen) and Razavi Khorasan (cities: Gonabad and Mashhad). The study timeline encompassed January 2020 through December 2021.

### 2.2 Study population and sampling strategy

The target population comprised COVID‐19‐positive patients undergoing dialysis treatment. In this study, all patients undergoing hemodialysis treatment from all centers of Mashhad and Gonabad (East of Iran) were included in the “Exposed” group. Then, according to the number of hemodialysis patients in each center and the target sample size, the number of non‐dialysis COVID‐19‐positive patients (“Nonexposed" group) for participating in the study was determined for each center (proportional sampling). Subsequently, patients were selected based on the list and simple random from each center, limited to those with PCR‐confirmed COVID‐19 infection. To control for age and gender variability, the nonexposed group was enlarged to include 316 patients, whereas the exposed group consisted of 104 individuals.

### 2.3 Inclusion and exclusion criteria

Inclusion criteria stipulated that patients must be adults (≥18 years), with confirmed SARS‐CoV‐2 infection through PCR testing, undergoing hemodialysis at the time of diagnosis; and receiving the treatment based on the protocol “Guidelines for the diagnosis and treatment of COVID‐19 disease at the levels of providing outpatient and inpatient services,” the 11th version, approved by the Ministry of Health and Medicine of Iran.

Exclusion criteria were meticulously defined to mitigate confounding variables. Patients were excluded if they had acute kidney injury, were under 18 years of age, or were hospitalized for COVID‐19 due to non‐renal etiologies. Additionally, those with coexisting pathologies such as COPD, malignancies, autoimmune diseases, or CKD, as well as kidney transplant recipients and users of immunosuppressive medication, were also excluded.

### 2.4 Data collection parameters

A comprehensive data collection framework was instituted, utilizing a structured checklist to compile clinical outcomes. This encompassed parameters such as mortality rates, changes in consciousness levels, ICU admissions, ventilator dependencies, and a spectrum of symptoms ranging from fever to malaise.

### 2.5 Variables and measurements

To ensure robust comparative analysis, a diverse set of laboratory indicators was included: hemoglobin (Hb), white blood cell (WBC), red blood cell (RBC), lymphocytes, CRP, ESR, blood pH, LDH, AST, INR, ALT, platelets, urea, potassium, sodium, calcium, and creatinine levels at admission time after diagnosis. Additionally, symptomatic presentation and critical care requirements were also captured.

### 2.6 Statistical analysis

Data were analyzed using SPSS version 22. Analytical techniques employed included independent sample *t*‐tests for normally distributed data, and analysis of variance for comparing means across multiple groups. All hypotheses were tested at a 95% confidence interval, with a *p*‐value < .05 considered statistically significant.

### 2.7 Ethical considerations

This investigation adheres to the principles of the Helsinki Declaration and was formally approved by the Institutional Review Board (IRB) of Birjand University of Medical Sciences, under the ethics code IR.BUMS.REC.1401.257.

## 3 FINDINGS

In this retrospective cohort study conducted at multiple centers in the cities of Birjand, Mashhad, Gonabad, Gaen, and Ferdows, a total of 420 COVID‐19 patients (confirmed by PCR) who were hospitalized during the years 2020−2021 were evaluated and followed‐up. Out of these, 104 patients with COVID‐19 were chronic dialysis recipients, and the control group consisted of 316 non‐dialysis COVID‐19 patients.

Based on Table 1, the two groups did not differ significantly in terms of gender (*p* = .091). The mean age was 64.81 ± 16.04 years in the dialysis group and 60.92 ± 17.89 years in the non‐dialysis group, which was not statistically significantly different.

**Table 1:** Comparison of baseline demographic variables between the two groups.

Variable		Dialysis (104 people)	Non‐dialysis (316 people)	*p* Value
Gender *N* (%)	Male	69 (66.3%)	180 (57.0%)	.091
	Female	35 (33.7%)	136 (43.0%)	
Average age (standard deviation)		64.81 (16.04)	60.92 (17.89)	.05

According to Table 2, in the assessment of clinical symptoms in patients, statistically significant differences were observed between the two study groups (*p* < .05). In the dialysis group, 39.4% of patients presented with altered consciousness, which was significantly higher compared to the non‐dialysis group (9.5%).

**Table 2:** Comparison of clinical symptoms at admission between the two groups.

Variable	Dialysis (104 people)	Non‐dialysis (316 people)	*p* Value
Altered consciousness	41 (39.4%)	30 (9.5%)	<.001
Dyspnea	69 (66.3%)	172 (54.4%)	.033
Headache	4 (3.8%)	70 (22.2%)	<.001
Lethargy	51 (49.0%)	150 (47.5%)	.781
Myalgia	11 (10.6%)	144 (45.6%)	<.0001
Anorexia	23 (22.1%)	129 (41.1%)	<.0001
Cough	33 (31.7%)	183 (57.9%)	<.0001
Fever	44 (42.3%)	149 (47.2%)	.39
Length of hospitalization (day) (SD)	7.73 (3.60)	5.20 (3.96)	<.0001

The most common symptoms upon admission in the dialysis group were dyspnea, lethargy, malaise, fever, and altered consciousness, in descending order. In the non‐dialysis group, the most prevalent symptoms included cough, dyspnea, lethargy, and malaise, in descending order.

The prevalence of flu‐like symptoms, including headache, malaise, anorexia, and cough, was higher in the non‐dialysis patients, while there were no statistically significant differences in the prevalence of fever, lethargy, and malaise between the two groups (*p* > .05).

The average length of hospitalization in the dialysis group was significantly higher than non‐dialysis group (*p* < .05).

**Figure 1:**
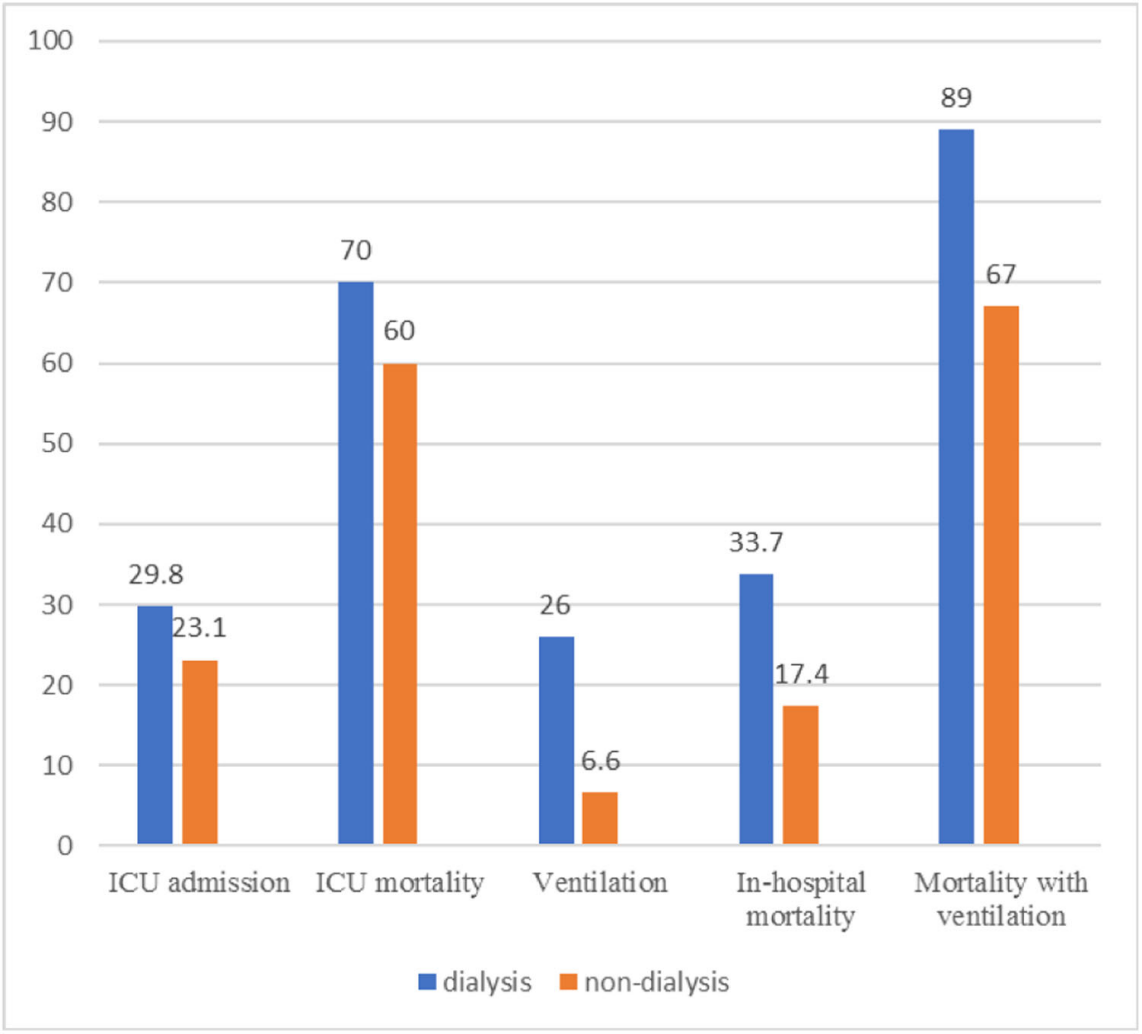
Comparison of disease outcomes between the two groups.

Figure 1 illustrates the disease outcomes in both groups, presented as percentages. Only in‐hospital mortality and the need for ventilation showed statistically significant differences between the two groups.

The mean laboratory variables in the two groups are shown in Table 3. Based on the statistical analysis results, there was a statistically significant difference between the mean values of WBC, RBC, Hb, platelets, sodium, potassium, calcium, urea, creatinine, blood pH, INR, ALT, LDH, ESR, and CRP in the two groups, such that the mean values of WBC, potassium, calcium, urea, creatinine, INR, ALT, ESR, and CRP were higher in the dialysis group, and the mean values of RBC, Hb, platelets, sodium, blood pH, and LDH were higher in the non‐dialysis group (*p* < .05). The laboratory variables lymphocyte count, AST, and CPK did not show statistically significant differences.

**Table 3:** Comparison of laboratory data at admission time after diagnosis between the two groups.

Laboratory variable	Non‐dialysis group (mean ± SD)	Dialysis group (mean ± SD)	*p* Value
WBC	7.55 ± 5.53	9.2 ± 6.79	.013
Lymphocyte	1320.67 ± 1779.46	1141.09 ± 853.02	.322
RBC	4.74 ± 0.66	3.78 ± 0.9	<.0001
Hb (g/dL)	13.37 ± 1.91	10.76 ± 2.48	
Platelet	181.68 ± 72.37	161.42 ± 72.46	.016
Sodium	137.26 ± 4.28	136.47 ± 13.43	.036
Potassium	4.15 ± 1.83	4.75 ± 1.01	.012
Calcium (mg/dL)	8.2 ± 0.59	8.5 ± 1.08	.033
Urea (mg/dL)	50.39 ± 43.39	124.8 ± 75.62	<.0001
Creatinine (mg/dL)	1.28 ± 0.63	5.38 ± 2.99	<.0001
Blood pH	6.89 ± 0.08	7.28 ± 0.13	<.0001
INR	1.10 ± 0.43	1.46 ± 1.21	<.0001
AST (u/L)	93.59 ± 360.91	91.67 ± 230.80	.08
ALT (u/L)	81.99 ± 345.39	87.57 ± 230.83	.01
CPK (u/L)	277.51 ± 597.013	217.09 ± 254.35	.64
LDH (u/L)	817.44 ± 1223.51	609.04 ± 413.21	.006
ESR (mm/h)	32.01 ± 22.57	56.25 ± 32	<.0001
CRP (mg/dL)	38.23 ± 23.27	67.44 ± 51.25	<.0001

Abbreviations: RBC, red blood cell; WBC, white blood cell.

According to Table 4, in the dialysis group, there was no significant relationship between patients' survival status and age, gender, or length of hospitalization. However, a significant association was found between admission to the ICU and the need for mechanical ventilation with patients' survival status (*p* < .001). Specifically, among the deceased patients, 62.85% required ICU admission, while only 13.04% of the survivors needed ICU admission. Furthermore, 68.57% of the deceased patients required mechanical ventilation during their hospitalization, whereas this percentage was only 4.34% among the survivors.

**Table 4:** Comparison of demographic variables and reported disease outcomes in the dialysis group based on mortality status.

Variable		Deceased (35 people)	Survivors (69 people)	*p* Value
Age	18−65	17 (48.57%)	29 (42.02%)	.33
	≥65	18 (51.43%)	40 (57.98%)
Gender	Male	22 (62.9%)	47 (68.1%)	.0592
	Female	13 (37.1%)	22 (31.9%)	
ICU admission	Yes	22 (62.85%)	9 (13.04%)	<.0001
	No	13 (37.15%)	60 (86.96%)	
Ventilator required	Yes	24 (68.57%)	3 (4.34%)	<.0001
	No	11 (31.43%)	66 (95.66%)	
Hospitalization length (SD)		7.36 (6.19%)	8.46 (7.38%)	.427

**Figure 2:**
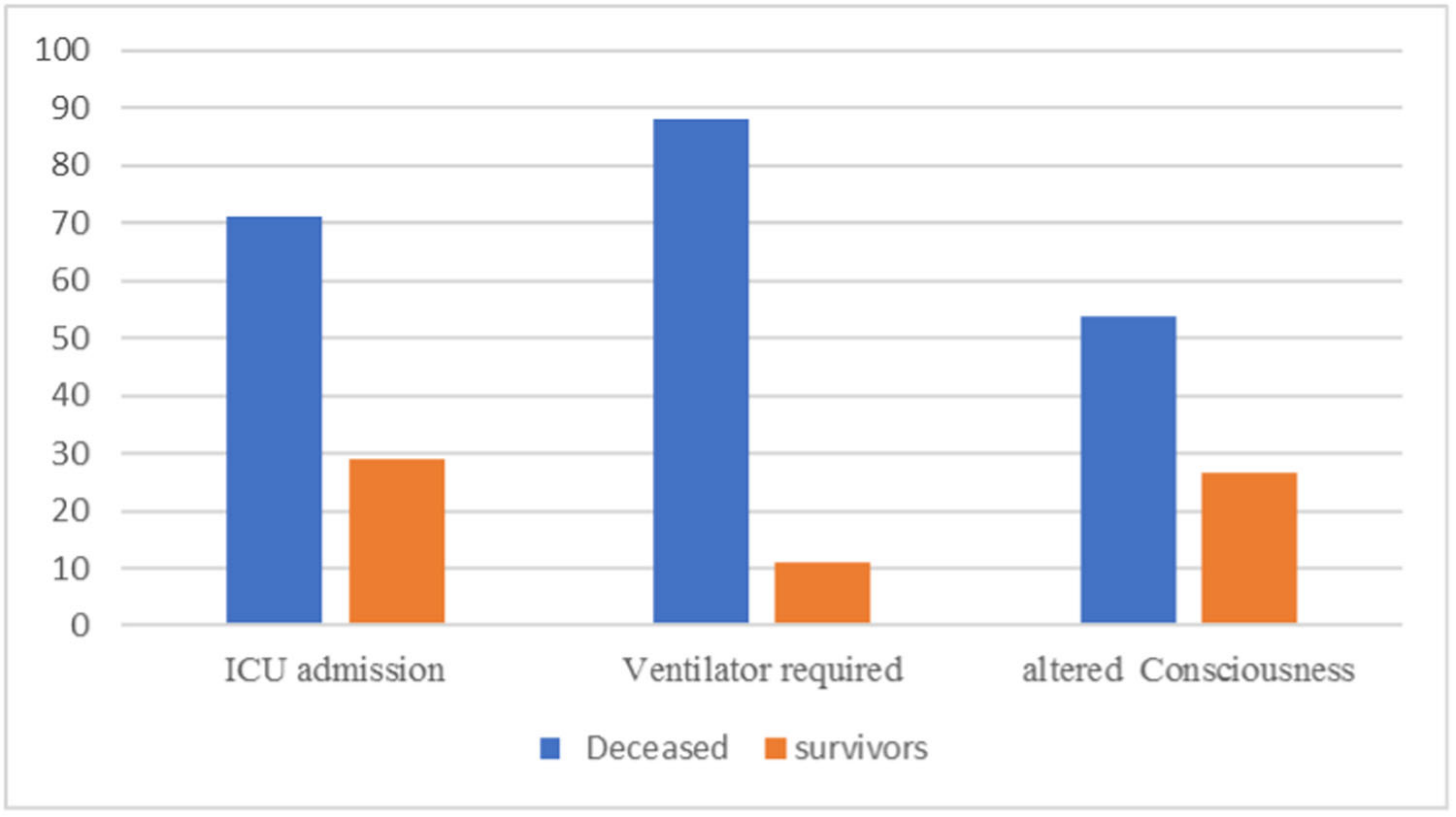
Comparison of reported outcomes and clinical sign of altered consciousness in the dialysis group based on mortality status.

Based on Figure 2, three variables, which were significantly different between survivors and deceased patients in the dialysis group, are compared in terms of percentages. According to this figure, deceased patients had significantly experienced decreased consciousness, required mechanical ventilation, and were admitted to the ICU.

**Figure 3:**
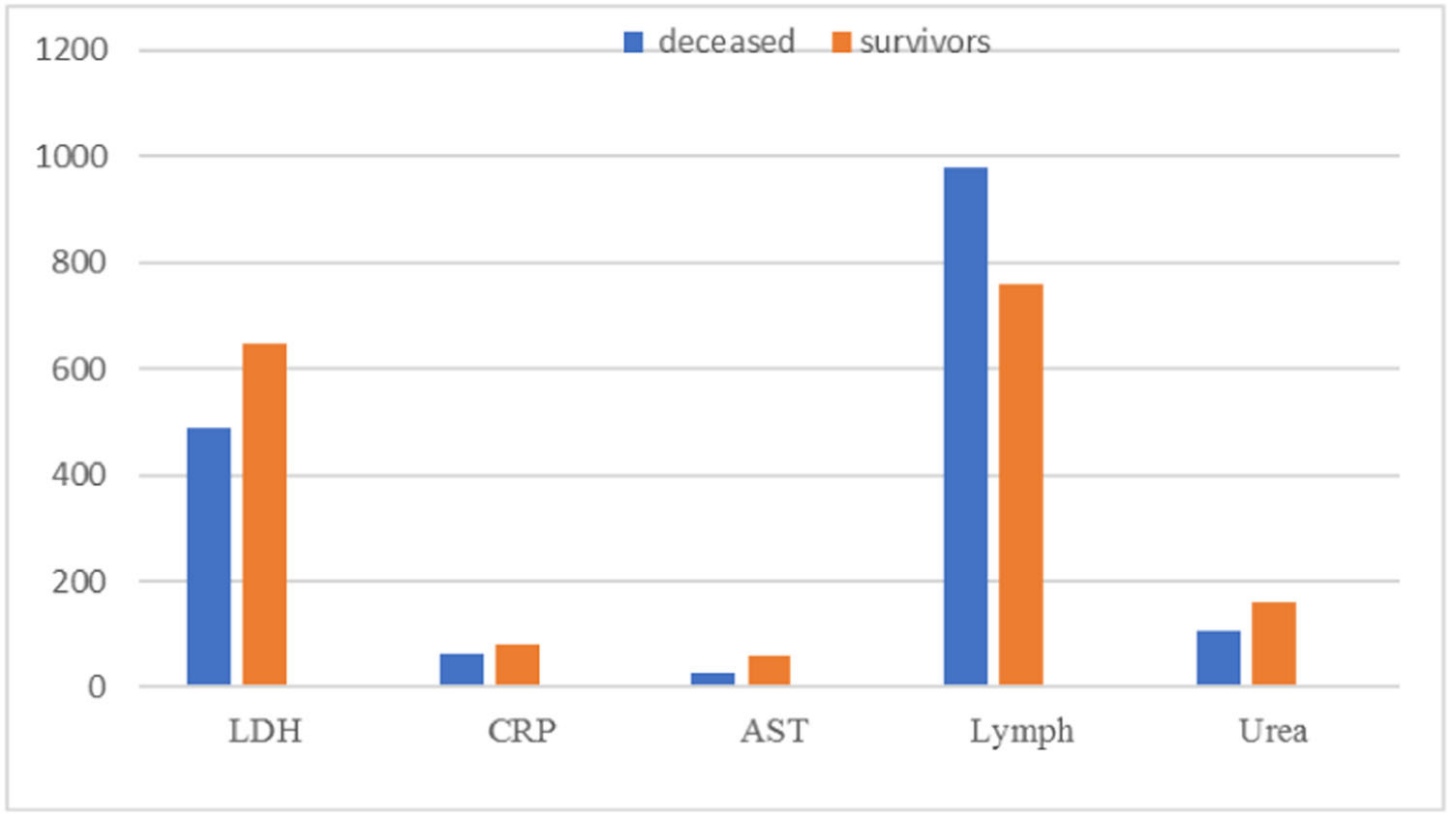
Comparison of laboratory data in the dialysis group based on mortality status.

According to Figure 3, the laboratory data in the dialysis group were compared based on mortality status. Based on this figure, due to the dispersed nature of the data for AST, LDH, and CRP variables, the median and Mann−Whitney test were used; based on this test, the levels of urea and AST were significantly different between the two groups (*p* < .05).

## 4 DISCUSSION

This study aimed to examine the severity and prognosis of COVID‐19 in patients with ESRD and compare it with non‐dialysis patients. According to the findings, there was no major difference in gender distribution and mean age between the two groups, but the dialysis group had a significantly higher percentage of seniors. Consistent with the findings of this study, Ng et al. also found that patients with kidney disease undergoing dialysis had a higher average age.^20^ The higher average age of ESRD patients than the general population and the increasing prevalence of this disease with age may explain this result.^21^

It was found in the study that the dialysis group had a greater number of symptoms of decreased consciousness and dyspnea. In contrast, the non‐dialysis group experienced a significant increase in symptoms like myalgia, anorexia, and cough, which are considered mild (cold‐like) symptoms. In the studies by Kenarkoohi et al. and Najafi et al., it was found that dyspnea was the most frequent clinical symptom in dialysis patients.^22^, ^23^ The high prevalence of this clinical symptom as an early admission sign may result from the late referral of dialysis patients to healthcare centers. In contrast, in the non‐dialysis group, the most common symptoms included cough, dyspnea, fever, and lethargy which are the most common initial clinical symptoms of COVID‐19.^24^ The difference in the pattern of clinical symptoms at admission in dialysis patients may be due to differences in the timing of hospitalization or changes in the severity of the disease in the dialysis group.

The dialysis group had significantly higher in‐hospital mortality; a significantly higher percentage of these individuals required mechanical ventilation, and the average length of hospitalization in this group was also significantly longer. Also, Ng et al. found that in‐hospital mortality and length of hospitalization were significantly higher in the dialysis group compared to the control group.^20^ The significant increase in mortality in dialysis patients with COVID‐19 may be due to a weakened immune system in these individuals, leading to the spread of infection and sepsis in these patients.^25^ On the other hand, the increased need for mechanical ventilation in dialysis patients may be due to an increased risk of respiratory complications resulting from underlying diseases or the late‐stage referral of these patients.

Based on laboratory data, the WBC, potassium levels, calcium levels, urea levels, creatinine levels, blood pH, INR, ALT, ESR, and CRP in the dialysis group were significantly higher, while the average RBC count, platelet count, Hb levels, sodium levels, and LDH levels were higher in the non‐dialysis group. In conjunction with the results of our study, Rastad et al. found that dialysis patients with COVID‐19 had significantly higher levels of creatinine, potassium, PT, ESR, and CRP.^17^ The considerable increase in creatinine and potassium levels in the dialysis group can be attributed to kidney dysfunction in these patients, while the elevation in PT may result from liver involvement and sustained inflammation in the dialysis group, potentially due to immune system alterations.^26^ The significantly lower levels of Hb and RBC in the dialysis group can be justified by the role of the kidneys in erythropoiesis and chronic anemias in kidney patients. Zuin et al. also reported similar findings in their study.^27^ They stated that CKD and hemodialysis increase the risk of anemia and the severity of COVID‐19 in these patients.^27^

Based on the research results, age and gender variables did not show a significant relationship with mortality in dialysis patients with COVID‐19. Consistent with our findings, Min et al. in their study also found that gender and age did not have a significant relationship with mortality in dialysis patients with COVID‐19.^28^ On the other hand, deceased dialysis patients had a significantly higher rate of ICU admission and ventilator use, indicating the severity of the disease in this group.^29^ Deceased dialysis patients also had a shorter length of hospitalization compared to survivors, although this difference was not statistically significant, which may suggest a rapid and aggressive course of the disease in these individuals. Various variables, including underlying cardiovascular diseases and diabetes, can be influential in predicting ICU admission, ventilator use, and length of hospitalization (23, 30, and 31).

In this study, 53.7% of deceased dialysis patients experienced a decrease in consciousness upon admission, which was significantly higher compared to survivors (*p* < .001). However, there was no significant difference in other, milder symptoms between the two groups (*p* > .05). Similar results were obtained in the Valeri study and the Min et al. study, where no significant differences in clinical symptoms were observed between deceased and surviving dialysis patients (these studies only investigated mild disease symptoms).^28^, ^30^ The significant increase in decreased consciousness in the deceased group may be attributed to the severity of COVID‐19 in these patients, which occurred as a result of delayed presentation for medical care after the onset of symptoms.

In the comparison of laboratory data between deceased and surviving dialysis patients, the deceased group had significantly higher WBC counts, urea levels, AST, and ALT levels, and blood pH in this group was significantly more acidic. Consistent with these results, the study by Shang et al. suggested that a significant increase in WBC count may indicate the severity of COVID‐19 in hemodialysis patients and contribute to increased mortality.^31^

Although in the present study, liver enzymes were significantly higher in the deceased group compared to survivors, in the study by Min et al., the average levels of liver enzymes such as AST and ALT in dialysis patients who died from COVID‐19 were lower compared to those who survived.^28^ SARS‐CoV‐2 positive CKD patients were found by Kumar et al. to have higher serum aminotransferase levels than their non‐CKD COVID‐19‐positive counterparts, and these levels were significantly correlated with calculated eGFR values. Thus, they concluded that elevated ALP values in CKD patients with COVID‐19 infection may be used as an indicator of declining kidney function.^32^ This contradiction between different studies needs more investigation in future studies. Generally, liver damage can be a result of underlying conditions in these individuals, COVID‐19 itself, or the therapeutic interventions performed on these patients.^33^ It should also be noted that liver damage may limit medical treatment choices made by the healthcare team, ultimately leading to the exacerbation of the disease in this group.^34^

This study also has limitations, such as not examining the role of vaccination on disease severity and prognosis, not examining the impact of COVID‐19 on patients with a milder form of the disease such as CKD and not considering risk factors such as hypoalbuminemia.

## 5 CONCLUSION

Based on the results of this study, it is possible to hypothesize a greater severity and worse prognosis of COVID‐19 in patients with ESRD. The presence of underlying diseases, including liver disorders, or more severe clinical symptoms such as decreased consciousness, can also be indicative of a worse prognosis in dialysis patients with COVID‐19. It is recommended to conduct further studies with a larger population at the provincial or national level. In this way, the role of more variables, including environmental, immunological, or racial factors and in the milder forms of the disease such as CKD, can also be investigated.

## AUTHOR CONTRIBUTIONS

**Tara Mohsenzadeh**: Conceptualization; data curation; investigation; methodology; writing—original draft; writing—review and editing. **Masood Ziaee**: Conceptualization; methodology; project administration; writing—original draft; writing—review and editing. **Hamid Salehiniya**: Data curation; project administration; formal analysis; writing—original draft; writing—review and editing. **Hossein Mohsenzadeh**: Conceptualization; investigation; methodology; validation; writing—original draft; writing—review and editing. **Amin Mirsani**: Conceptualization; data curation; investigation; project administration; writing—original draft; writing—review and editing. **Vajehallah Raeesi**: Conceptualization; investigation; project administration; supervision; validation; writing—original draft; writing—review and editing.

## CONFLICT OF INTEREST STATEMENT

The authors declare no conflict of interest.
